# Association between Objectively Determined Physical Activity Levels and Body Composition in 6–8-Year-Old Children from a Black South African Population: BC–IT Study

**DOI:** 10.3390/ijerph18126453

**Published:** 2021-06-15

**Authors:** Caroline M. Sedumedi, Xanne Janssen, John J. Reilly, Herculina S. Kruger, Makama Andries Monyeki

**Affiliations:** 1 Physical Activity, Sport and Recreation Research Focus Area, Faculty of Health Sciences, North-West University, Potchefstroom 2520, South Africa; mmalekgotlha@gmail.com; 2 Physical Activity for Health Group, School of Psychological Sciences and Health, University of Strathclyde, Glasgow G1 1QE, Scotland, UK; xanne.janssen@strath.ac.uk (X.J.); john.j.reilly@strath.ac.uk (J.J.R.); 3 Centre of Excellence for Nutrition, North-West University, Potchefstroom 2520, South Africa; Salome.Kruger@nwu.ac.za

**Keywords:** fat mass, fat free mass, deuterium dilution method, objective, physical activity

## Abstract

Physical inactivity in children is a global pandemic in parallel with increasing obesity prevalence. However, studies assessing the association between physical activity (PA) and body composition (BC) report conflicting findings, possibly because of the different methodologies across studies, with objective methods promising reliable results. This study determines the association between objectively determined PA levels and BC in 6–8-year-old children from a black South African population. Ninety-three children aged 6–8 years, who formed part of a larger study on BC using the deuterium dilution method (DDM), were included. Height and weight were measured according to the standard procedures, and body mass index z-score was calculated. Fatness was determined by DDM. An accelerometer was used to measure PA levels. Regression models were performed to determine the relationship between PA and BC. Approximately 23% of the children did not meet the recommended PA guidelines and 27% were overfat. After adjustments were made, more time spent in vigorous PA was significantly associated with lower fat mass (β = −0.25, *p* = 0.01, 95%CI: −11.08; −1.20) and fat mass % (β = −0.20, *p* = 0.04, 95%CI: −12.63; −0.18). Participation in high PA, especially of high intensity, was associated with reduced adiposity in children. Behavioural changes such as increasing high intensity PA is strongly recommended for reducing adiposity.

## 1. Introduction

The decline in physical activity (PA) levels in children has been linked to a high prevalence of obesity and overweight [1,2,3]. In recent regional studies in South African children and adolescents, a high prevalence of obesity and physical inactivity were reported [4,5]. In sub-Saharan Africa, the prevalence of overweight and obesity increases over time during childhood and is more prevalent in girls compared to boys [6,7]. If overweight and obesity are not identified early in life and preventative measures are not put in place, physical inactivity and excess adiposity in childhood can have long-lasting negative health effects [5,6,7,8,9,10]. Furthermore, childhood obesity is associated with an increased risk of morbidity and mortality later in life—it accounts for as much as 20% of cardiovascular deaths in midlife [11]. To achieve health benefits, it is recommended that children and adolescents aged 5–17 years participate in an average of 60 min or more moderate-to-vigorous physical activity (MVPA) per day [12]. Although the benefits of PA have been well identified, physical inactivity remains a major public health problem [13,14]. The Healthy Active Kids South Africa (HAKSA) 2018 report indicated that 50% of children are not meeting the recommended average of 60 min of MVPA per day [15]. It appears that the adoption of urban lifestyles, introduction of technology-related devices and the shift from active transport, particularly in children from rural areas, are all responsible for decreasing levels of PA [2,16].

In population-based studies, it is convenient to use self-reporting methods of PA and body mass index (BMI) as a proxy for adiposity, as these are simple and affordable methods [17,18,19]. However, studies with self-reported PA and the use of BMI often report inconclusive findings. Even though BMI correlates with body fat percentage [20], it cannot distinguish between fat mass (FM) and fat free mass (FFM) [21]. Excessive fat accumulation is associated with obesity-related pathology and morbidity [22]. Additionally, although the use self-reported or parental-reported PA is easy and cost effective, this method is subject to participants’ recall bias and, as such, often fails to report the true levels of PA [23,24]. The objective assessment of PA and adiposity is warranted for valid results. Campa and colleagues [25] in their review study on available methods for assessing body composition alluded to the importance of monitoring and appropriately assessing body composition to be crucial for accurate evaluation.

Studies assessing the association between PA and body composition (BC) have been extensively performed, but findings are contradictory [26,27,28,29]. What is currently known about the association between objective PA and BC is largely from high income countries, and data on low to middle income countries are scanty. While few studies have been carried out in South African children, the focus has not been on the years of school entry [30,31,32,33,34]. PA levels decrease by up to 50% in the years of school entry; furthermore, overweight and obesity are higher in mid-childhood [35,36,37]. More studies to understand the association between sedentary behaviour (SB), PA and adiposity in South African school children are urgently needed [30]. We attempt to minimise this gap by reporting the association between PA by an accelerometer and BC by stable isotope methods in South African children in the years of school entry. Findings from the present study add to the scanty data on the effect of PA on BC in children from sub-Saharan Africa that employ methods of higher clinical validity. Furthermore, the results can guide future studies on the use of PA and BC measures. It was therefore fitting to investigate the association between objective PA levels and BC determined by stable isotope methods in 6-to-8-year-old South African children. We hypothesised that higher levels of PA are associated with lower body fatness in South African children.

## 2. Methodology

### Study Design and Participants

The current study follows a cross-sectional design and is descriptive in nature. It forms part of a larger study on BC using the isotope technique (BC–IT) [5]. The larger study examines the relationship between objective (stable isotope) and indirect (anthropometric variables) measures of BC indices and objective (accelerometer), and subjective (physical activity questionnaire for older children (PAQ-C)) measures of PA among 6-to-8-year-old South African children, and the relationships with other health-related determinant factors (blood pressure, lifestyle habits). For the present study, all children with ActiGraph and deuterium dilution method (DDM) data (93 children: 51 girls and 42 boys) attending different primary schools within the Tlokwe Local Municipality of the North West province were included. The Generalised Linear Model for Analysis of Variance was used to calculate the statistical power for the appropriate sample size for a power of 0.80 and α-level of 0.05 at a CI of 95%. The analyses were carried out for FM, with sex used as a factor, and the Leven’s test of Equality of Error Variances based on the adjusted median and with adjusted degree of freedom (df2 = 88.57; *p* = 0.05) showed that the null hypothesis tested was equal across the group. Then, statistical power between subject effects was 0.83. For posterior analysis, we used the independent samples test of Bayersian statistics to examine the difference in means of FM% as a continuous variable across two levels or sex (boys = 1 and girls = 2) groups of a categorical variable. We obtained the 95% credible interval which told us that we could be 95% certain that the mean difference in FM% is between 3.7968 and 8.7832; as our mean difference is 6.29, we could be confident that this difference is an accurate reflection of the sample. From the Bayesian analysis for posterior distribution, we can state that the most likely difference between mean FM%s was 6.29; however, our BF (Bayes Factor) = 0.0, which told us that the null is a more probable explanation for the data than the alternate. In other words, the difference in mean FM% between boys and girls was not significant.

The study was approved by the Health Research Ethics Committee (HREC) in the Faculty of Health Sciences of North-West University (ethics no: NWU-00025-17-A1). After advertisement to primary schools with permission from the Department of Education, approval was received from school principals. The class lists for Grade R to Grade 3 were requested from the five participating schools. From the lists, every third child was nominated to participate in the study. Subsequently, only children whose parents gave informed consent were included in the study. Children were asked to give verbal assent in the case of 6-year-olds and written consent in 7–8-year-olds before measurements could commence.

## 3. Measuring Instruments

### 3.1. Socio-Demographic Questionnaire

Socio-demographic information was collected by use of a socio-demographic questionnaire; the information was used to put schools into different quintiles according to socio-economic status. The Quintiles 1 to 3 refer to schools in the most economically disadvantaged (poorest) geographical areas (non-fee-paying schools that receive more funding per learner from the government), and for statistical analysis, they were coded as number 1. Quintiles 4 and 5 refer to fee-paying schools that require less governmental support, because parents can afford to pay fees [38,39], and for statistical analysis, they were coded as number 2.

### 3.2. Anthropometric Measurements

Anthropometric measures of height (cm) and weight (kg) were determined by following the International Society for the Advancement of Kinanthropometry (ISAK) procedures [40]. Measurements were taken by level I anthropometrists. To ensure privacy, anthropometric measurements were taken in separate rooms for boys and girls. A Harpenden portable stadiometer (Holtain Limited, Crosswell, Crymych, UK) was used to measure height to the nearest 0.1 cm with the child barefoot and standing upright with the head in the Frankfort plane. Weight was measured to the nearest 0.1 kg with an electronic scale (Beurer Ps07 Electronic Scale, Ulm, Germany), with participants wearing minimal clothing and no shoes.

Two measurements were taken of each variable indicated, and the average of the two measurements was used for the analyses. Weight and height were used to calculate BMI (weight/height^2^); BMI z-score was calculated relative to WHO reference data [41].

## 4. Body Composition by Deuterium Dilution Method

BC was determined by use of the DDM. Sample collection and analysis followed the protocol provided by the International Atomic Energy Agency (IAEA). The concept and methodology are explained in detail elsewhere [4,42,43]. TBW was measured using a dilution of deuterium oxide (99.8%) sterility tested. Each child provided a saliva sample after an overnight fast, referred to as the pre-dose saliva sample. Then, each child received a dose of deuterium oxide-labelled water that was prepared according to age and sex. To avoid spilling, drinking straws were used, and bottles were rinsed twice with drinking water. Post-dose saliva samples were collected at 2 and 3 h. After the collection of the entire saliva sample from a participant, each child received a juice drink and small snack. The saliva samples were stored at −20 °C in the lab until analysis was performed. Fourier transform infrared (FTIR) spectroscopy (FTIR 4500t spectrophotometer, Agilent) was used to analyse the saliva samples. Age- and sex-specific Lohman hydration factors for children were used to calculate FFM, FM and FM% [4,42,43]. The children were classified as underfat, overfat, and obese at the <2nd, >85 to 95th and >95th centiles of body fat percentage, respectively [44,45,46].

## 5. Physical Activity Using ActiGraph Accelerometer

PA and SB were assessed using the ActiGraph accelerometer (Model GT3X-BT, Fort Walton Beach, FL, USA), which has been validated for use in children [47]. Trained research assistants fitted ActiGraphs on elastic belts worn at the waist (just over the right mid-axillary line), according to the manufacturer’s instructions. Participants were instructed to wear the ActiGraph for a minimum of 10 h/day for 7 consecutive days. They were advised to remove the accelerometers only during water-based activities such as bathing or swimming and when going to bed. Each child, with the help of the parents/guardian, was required to complete a daily log sheet indicating the time the Actigraph was worn and removed. An instruction manual on the proper usage of accelerometers was given to each participant for additional guidance.

Following the final day of data collection, accelerometers were returned to the school. ActiLife software (Version 6.13.3) was used to extract and analyse data. PA data are expressed as average daily minutes spent in light PA (LPA) ≥ 100 counts per minute, moderate PA (MPA) ≥ 2296 counts per minute and vigorous PA (VPA) ≥ 4012 counts per minute. Average daily minutes spent in SB are also reported (<99 counts per minute) [48]. Time in MVPA was calculated as the sum of MPA and VPA [33]. Participants who provided a minimum of 4 days of valid data, including 1 weekend day, were included in the analysis. Valid days were those days in which the accelerometer was worn for at least 600 min (10 h) per day. Consecutive zero counts for 20 min or more was considered as non-wear time [48].

## 6. Statistical Analysis

Statistical Package for Social Science (SPSS, Version 27; IBM Corp., Armonk, NY, USA) was used to analyse data. To describe participant characteristics, means and standard deviations were computed for the whole group and for girls and boys separately. Normality was assessed using normal QQ plots for visual inspection and the statistical one-sample Kolmogorov–Smirnov normal distribution examination. Data that were not normally distributed were log transformed. To describe gender differences, the independent sample *t*-test was performed for normally distributed data and Mann–Whitney U tests when data were not normally distributed. To achieve the aim of determining the relationship between PA and BC, multiple linear regression models were used to determine the associations between PA (SB, LPA, MPA, VPA and MVPA) and BC variables (BMI, FM, FFM, FM%). The outputs are reported as standardised β coefficients, p-value and standardized r-square (r^2^). The crude model and adjusted models were reported, and models were adjusted for age, sex, and school quintile: Model 1 = sex; Model 2 = age; Model 3 = school quintile.

## 7. Results

Table 1 shows descriptive statistics of participants, reported as means and standard deviations. Girls were significantly (*p* < 0.05) more sedentary and had higher levels of fat mass compared with boys, when using objective measures. No significant (*p* > 0.05) gender differences were reported when BMI z-scores were used. Boys spent significantly (*p* < 0.05) more time in MPA and VPA than girls. There was no significant (*p* > 0.05) gender difference in time spent in LPA.

Table 2 reports the prevalence of weight and PA categories, based on DDM, and shows that 27% of the children were classed as having a high FM%. There was a noted significant gender difference in the prevalence of high fatness; more girls (41.2%) had high FM% compared with boys (9.5%; *p* < 0.001). When BMI z-scores were used to assess BC, no gender differences were noted. A total of 77.4% of children achieved the recommended average of 60 min per day of MVPA. A significantly higher number of boys (97.6%) met the guidelines compared with girls (60.8%).

Table 3 shows that children who did not meet the MVPA guideline had significantly higher FM, FM% and FMI compared with those who met the MVPA guideline.

Table 4 presents the crude and adjusted regression models to assess the association between PA levels and BC variables. Generally, correlation coefficients between PA and BC are very low. In the crude model, time spent in VPA (β =−0.28; *p* = 0.01, 95%CI: −7.07; −1.20), MVPA (β = −0.25; *p* = 0.01, 95%CI: −11.08; −1.20) and MPA (β = −0.25; *p* = 0.01, 95%CI: −11.08; −1.20) were associated with lower FM and FM%. After adjustments were made, time spent in VPA remained significantly associated with lower FM (β = −0.25; *p* = 0.01, 95%CI: −11.08; −1.20) in the age-adjusted model and with lower FM% (β = −0.20; *p* = 0.04, 95%CI: −12.63; −0.18) in the gender-adjusted model. Higher MVPA showed a trend of an association with lower FM in the sex- and school quintile-adjusted model; the association was, however, not significant (*p* = 0.06). No significant association was reported between PA and BMI z-scores (*p* > 0.05).

## 8. Discussion

The current study, with the aim of determining the relationship between objectively measured PA and BC, found that in line with our expectations, high PA levels were associated with low body fatness. This association was only observed when BC was directly assessed by use of DDM. The use of BMI z-scores as a proxy for adiposity did not yield any significant association. Children who did not meet the recommended MVPA levels had significantly higher adiposity compared with those who met the guidelines. Girls were more overweight and obese compared with their male counterparts. The prevalence of children who meet the recommended average of 60 min per day of MVPA was 77.4%, and more boys than girls met the guidelines.

Although not enough data exist to set the global threshold for time spent in SB or screen time, evidence does suggest that more time spent in SB is associated with adverse health risk factors [12]. It has been reported that children from South Africa and Kenya spend most of their time in SB and LPA [49,50,51,52]. In the current study, girls spent an average of almost 6 h per day in SB; this was compared to 5½ h spent by boys. Previous studies have reported that spending more time in SB is associated with unfavourable BC indicators in children [53,54]. Furthermore, it has been reported that MVPA levels did not influence the relationship between SB and adiposity in children [53]. This means that spending more time in SB can have detrimental health effects even if recommended MVPA levels are achieved. The results of the current study only show a trend of a positive relationship between SB and FFM which was, however, not significant. This may be as a result of the small sample size. Interventions are needed to reduce SB and replacing SB with high intensity activity has been reported to have positive health outcomes [55].

Consistent with other studies in sub-Saharan Africa, children spend more time in LPA than in MVPA [50,56,57]. Evidence of whether LPA has beneficial effects on BC is scanty and more research on this is needed [50,55]. The current study does not report any significant relationship between LPA and BC. In line with our hypothesis, spending more time in MVPA was associated with reduced risk of obesity (FM and FM%). These findings are in accordance with findings from numerous studies. Cross-sectional studies, alike with longitudinal studies, have revealed that high intensity PA is associated with reduced odds of obesity [27,29,33,58]. A study carried out in 7-year-old children from Spain revealed that accelerometer-assessed MVPA was associated with lower FM [59]. Additionally, research conducted in children aged 6–8 years old from Finland reported that high levels of LPA and MVPA were associated with low levels of FM index; vigorous PA showed the highest magnitude of the association [27]. Although it is generally suggested that MVPA is associated with reduced fatness, it appears that VPA plays the bigger role since it is associated with larger energy expenditure [60,61]. In the present study, high volumes of VPA were associated with reduced FM and FM%, and this finding is confirmed by a study performed by Dencker and colleagues [62].

Although the beneficial effect of high MVPA on BC has been well documented, not all findings agree. Vanderloo and colleagues [24] could not show that high PA levels were associated with reduced body fatness. The reason no association was found could be because of the self-reporting of PA in the group of children, which may not have been accurate [63,64]. In children from Tunisia, no significant association could be found between self-reported PA (PAQ-C) and BC, whereas objectively measured PA showed an inverse significant relationship with adiposity in the same study. The reason no association was found when self-reported PA was used was probably due to the reported poor level of agreement between PA measured by PAQ-C and the accelerometer [63]. Hence, it could conceivably be hypothesised that in populations where self-reports are not in agreement with objective measures, beneficial effects of PA will not be seen if self-reported PA is used. Self-reports and objective measures classify children’s PA levels differently in different settings [56,63]. This issue has grown in importance, considering the detrimental effect of both physical inactivity and excessive fatness if left untreated. The inconsistency in some studies is also in part due to the use of BMI as a measure of adiposity. A study in 6–8-year-old children from the United Kingdom reported that higher levels of PA were associated with low FM determined by DDM but could not find any significant associations when adiposity was reported as BMI [65]. Likewise, we did not pick up any significant relationship between PA and BMI z-scores. This implies that the use of BMI z-scores as a proxy for adiposity in this population masks the beneficial effect of PA on BC and should be used with caution. In vivo methods of BC assessment such as densitometry, DDM, bioelectrical impedance, conductance and dual-energy X-ray absorptiometry are proven to be more accurate with a higher clinical validity [21,66]. As such, the use of high-quality measures, i.e., objective measures of PA and direct measures of BC, are needed in South African children.

We provide evidence that there is a significant difference in adiposity between children who meet the recommended MVPA levels and those that do not. Children who do not achieve recommended MVPA levels have significantly higher levels of adiposity. This highlights the beneficial effect of achieving the recommended PA levels on the prevalence of obesity. The current study reports that over 70% of children achieve recommended levels of PA. Although these levels are higher than those reported in other studies [67,68,69], almost a third of these children have excessive fatness. Intervention strategies are needed to increase high-intensity PA. Emphasis should also be put on diet and other variables that might have an impact on childhood adiposity [11]. However, with a small sample size, caution must be applied when interpreting these findings. The study does, however, shed light on the role that PA plays on the development of childhood obesity.

*Strengths and limitations*: The strength of the current study is the use of high-quality methods to record PA levels. Furthermore, adiposity was determined by use of DDM, which is a highly accurate ‘reference’ method of measuring BC [65]. The limitation is the cross-sectional design, and a small sample size that is not a representative sample of the children in the province. Furthermore, the study only focused on predominately Setswana-speaking children. Future studies should include a larger sample size that is representative of the children from the North West province, with all ethnicities represented. Longitudinal studies are also needed to assess the causal nature of any associations identified. We also recommend intervention studies geared towards the reduction in physical inactivity and obesity in children.

## 9. Conclusions

In line with our hypothesis, it can be concluded that participation in PA, especially of high intensity, decreased the likelihood of obesity in this sample of predominantly Setswana-speaking children. Furthermore, not achieving the recommended average of at least 60 min per day of MVPA was associated with a higher adiposity. Advocacy for behavioural changes such as increasing high intensity PA is warranted.

## Figures and Tables

**Table 1:** Descriptive statistics of participants.

Variable	Whole Group (*N* = 93)	Girls (*n* = 51)	Boys (*n* = 42)	
	Mean ± SD	Mean ± SD	Mean ± SD	*p*-Value
Age (yr)	7.7 ± 1.3	7.7 ± 1.3	7.6 ± 1.2	0.89
Height (cm)	121.6 ± 8.7	121.4 ± 9.0	121.7 ± 8.3	0.86
Weight (kg)	23.9 ± 5.9	24.6 ± 6.5	23.4 ± 5.1	0.45
BMI z-scores	−0.1 ± 1.1	−0.1 ± 1.1	−0.2 ± 1.0	0.20
SB (min)	342 ± 52	356 ± 49	325 ± 49	0.01
LPA (min)	363 ± 45	355 ± 44	372 ± 43	0.06
MPA (min)	57 ± 16	49 ± 13	66 ± 15	<0.01
VPA (min)	23 ± 11	19 ± 9	27 ± 11	<0.01
MVPA (min)	80 ± 25	67 ± 20	93 ± 23	<0.01
FM (kg)	6 ± 3	7 ± 3	5 ± 2	0.04
FFM (kg)	17 ± 3	17 ± 4	18 ± 3	0.19
FM%	26 ± 7	28 ± 7	22 ± 5	<0.01

SD = standard deviation; BMI = body mass index; SB = sedentary behaviour; LPA = light physical activity; MPA = moderate physical activity; VPA = vigorous physical activity; MVPA = moderate-to-vigorous physical activity; FM = fat mass; FFM = fat free mass; yr = year; cm = centimetre; kg = kilogram; min = minutes; % = percent.

**Table 2:** Prevalence of weight and physical activity categories.

Categories	All, *n* (%) 93	Girls, *n* (%) 51	Boys, *n* (%) 42	*p*-Value
**BMI z-scores**				
Underweight	9 (10)	5 (10)	4 (9.5)	0.67
Normal weight	72 (77)	38 (75)	34 (81.0)	
Overweight/obese	12 (13)	8 (16)	4(9.5)	
**DDM**				
Underfat	11 (12)	1 (2.0)	10 (24)	<0.01
Normal fat	57 (61)	29 (56.9)	28 (67)	
Overfat	25 (27)	21 (41.2)	4 (9)	
**MVPA**				
≥60 min MVPA	72 (77)	31 (61)	41 (98)	<0.01
<60 min MVPA	21 (23)	20 (39)	1 (2)	

BMI = body mass index; DDM = deuterium dilution method; MVPA = average moderate-to-vigorous physical activity; min = minutes.

**Table 3:** Descriptive characteristics of participants according to the moderate-to-vigorous physical activity categories.

	MVPA < 60 min/day	MVPA ≥ 60 min/day	*p*-Value
Variables	Mean (SD)	Mean (SD)	n (SD)
Weight (kg)	25.71 (±7.56)	23.41 (±5.22)	0.11
BMI z-scores	0.28 (±1.25)	−0.15 (±0.98)	0.11
FFM (kg)	17.90 (±4.40)	17.37 (±3.03)	0.52
FM (kg)	7.81 (±3.95)	6.05 (±2.79)	0.02
FM%	29.09 (±7.78)	24.98 (±6.25)	0.01

BMI = body mass index; FM = fat mass; FFM = fat free mass; FM% = fat mass percentage; MVPA = moderate-to-vigorous physical activity; kg = kilograms; SD = standard deviation; % = percent.

**Table 4:** Associations between body fatness components and physical activity variables.

	BMI z-Score				FM				FM%				FFM			
	r^2^	β	*p*	95%CI	r^2^	β	*p*	95%CI	r^2^	β	*p*	95%CI	r^2^	β	*p*	95%CI
**SB (Crude)**	−0.01	−0.05	0.59	−4.11; 2.36	0.01	0.14	0.17	−2.92; 16.22	0.01	0.16	0.13	−4.62; 36.52	0.00	0.10	0.31	−4.99; 15.52
Model 1	0.01	−0.11	0.33	−5.01; 1.72	0.07	0.06	0.57	−6.91; 12.54	0.20	0.02	0.82	−17.20; 21.73	0.02	0.16	0.14	−2.62; 18.65
Model 2	0.07	−0.17	0.11	−6.03; 0.63	0.16	−0.03	0.90	−10.06; 8.83	0.02	0.11	0.33	−11.25; 32.75	0.55	−0.14	0.06	−8.85; 14.43
Model 3	−0.01	−0.06	0.58	−4.25; 2.41	0.03	0.10	0.32	−4.79; 14.63	0.04	0.11	0.27	−9.26; 32.21	−0.01	0.11	0.29	−4.92; 16.19
**LPA (Crude)**	0.02	0.17	0.10	−0.67; 7.35	−0.01	−0.02	0.82	−13.55; 10.72	−0.01	0.01	0.94	−25.13; 27.18	0.01	−0.14	0.19	−21.32; 4.33
Model 1	0.10	0.19	0.06	−0.05; 8.05	0.07	0.03	0.74	−9.90; 13.88	0.21	0.10	0.28	−10.80; 36.45	0.02	−0.17	0.11	−23.51; 2.41
Model 2	0.03	0.13	0.001	1.90; 10.01	0.18	0.15	0.14	−3.01; 20.42	0.02	0.08	0.44	−16.96; 38.36	0.54	0.14	0.06	−0.32; 18.47
Model 3	0.01	0.17	0.10	−0.68; 7.38	0.02	−0.02	0.88	−12.92; 11.04	0.03	0.02	0.86	−23.35; 27.86	−0.00	−0.14	0.19	−21.45; 4.37
**MPA (Crude)**	−0.01	−0.07	0.51	−2.27; 1.14	0.05	−0.25	0.01	−11.08; −1.20	0.08	−0.30	0.003	−26.61; −5.67	−0.01	−0.03	0.75	−6.31; 4.57
Model 1	−0.00	0.00	0.99	−1.99; 1.99	0.08	−0.13	0.25	−9.00; 2.36	0.20	−0.09	0.40	−16.24; 6.55	0.01	−0.21	0.25	−9.97; 2.59
Model 2	0.05	−0.02	0.81	−1.89; 1.48	0.19	−0.18	0.06	−9.12; 0.11	0.09	−0.28	0.18	−1.85; 0.36	0.52	0.09	0.20	−1.34; 6.21
Model 3	−0.02	−0.08	0.49	−2.47; 1.19	0.05	−0.21	0.06	−10.36; 0.19	0.09	−0.25	0.19	−1.64; 0.32	−0.02	−0.04	0.71	−6.95; 4.75
**VPA (Crude)**	0.02	−0.18	0.07	−1.92; 0.09	0.07	−0.28	0.01	−7.07; −1.20	0.11	−0.35	0.001	−17.28; −4.93	−0.01	0.02	0.87	−3.00; 3.52
Model 1	0.02	−0.16	0.16	−1.87; 0.32	0.10	−0.20	0.07	−6.03; 0.23	0.23	−0.20	0.04	−12.63; −0.18	−0.00	−0.04	0.71	−4.19; 2.85
Model 2	0.07	−0.17	0.10	0.04; 0.38	0.22	−0.25	0.01	−6.34; −0.96	0.13	−0.34	0.12	−1.90; 6.21	0.52	0.07	0.30	−1.06; 3.43
Model 3	0.01	−0.19	0.07	−0.17; 0.13	0.08	−0.25	0.15	−6.69; −0.76	0.13	−0.32	0.08	−0.11; 1.72	−0.02	0.01	0.89	−3.10; 3.58
**MVPA (Crude)**	−0.01	−0.07	0.51	−2.27; 1.14	0.05	−0.25	0.01	−11.08; −1.20	0.08	−0.30	0.003	−26.62; −5.67	−0.01	−0.03	0.75	−6.31; 4.57
Model 1	−0.00	0.00	0.99	−1.99; 1.99	0.08	−0.13	0.25	−9.00; 2.36	0.20	−0.09	0.40	−16.24; 6.55	0.01	−0.14	0.25	−9.97; 2.59
Model 2	0.05	−0.02	0.81	−1.89; 1.48	0.19	−0.18	0.06	−9.12; 0.11	0.09	−0.28	0.18	−0.36; 1.82	0.52	0.09	0.20	−1.34; 6.21
Model 3	−0.02	−0.08	0.49	−2.47; 1.19	0.05	−0.21	0.06	−10.36; 0.19	0.09	−0.25	0.19	−0.32; 1.64	−0.02	−0.04	0.71	−6.95; 4.75

Model 1 = sex; Model 2 = age; Model 3 = school quintile; 95% CI = 95% confidence interval; BMI = body mass index; SB = sedentary behaviour; LPA = light physical activity; MPA = moderate physical activity, VPA = vigorous physical activity; MVPA = moderate-to vigorous physical activity; FM = fat mass; FFM = fat free mass.

## Data Availability

The data presented in this study are available on request from the corresponding author. The data are not publicly available due to privacy principles and NWU data sharing policy.
